# Mitochondrial Lipid Signaling and Adaptive Thermogenesis

**DOI:** 10.3390/metabo11020124

**Published:** 2021-02-22

**Authors:** Helaina Von Bank, Mae Hurtado-Thiele, Nanami Oshimura, Judith Simcox

**Affiliations:** Department of Biochemistry, University of Wisconsin-Madison, Madison, WI 53706, USA; hcvonbank@wisc.edu (H.V.B.); hurtadothiel@wisc.edu (M.H.-T.); oshimura@wisc.edu (N.O.)

**Keywords:** brown adipose tissue, thermogenesis, mitochondria, lipids, glycerolipids, cardiolipin, acylcarnitine, free fatty acid, plasmalogen, ketone

## Abstract

Thermogenesis is an energy demanding process by which endotherms produce heat to maintain their body temperature in response to cold exposure. Mitochondria in the brown and beige adipocytes play a key role in thermogenesis, as the site for uncoupling protein 1 (UCP1), which allows for the diffusion of protons through the mitochondrial inner membrane to produce heat. To support this energy demanding process, the mitochondria in brown and beige adipocytes increase oxidation of glucose, amino acids, and lipids. This review article explores the various mitochondria-produced and processed lipids that regulate thermogenesis including cardiolipins, free fatty acids, and acylcarnitines. These lipids play a number of roles in thermogenic adipose tissue including structural support of UCP1, transcriptional regulation, fuel source, and activation of cell signaling cascades.

## 1. Introduction

Body temperature regulation is a selective advantage that has allowed endotherms to thrive in diverse climates. Heat production can occur through shivering and nonshivering thermogenesis; nonshivering thermogenesis primarily occurs in the brown and beige adipocytes [1]. One of the major mechanisms of heat production in these thermogenic adipocytes is through uncoupling protein 1 (UCP1), which facilitates the diffusion of protons into the inner mitochondria without coupling the proton mobility to ATP synthase. The potential energy of the proton gradient is converted to heat as the protons diffuse into the inner mitochondria [2]. Other mechanisms of thermogenesis include futile cycles of calcium, phosphocreatine, and free fatty acids [3].

Due to the dissipation of the proton gradient and futile cycles, thermogenesis is energy demanding and has been an area of intense study for body weight control and metabolic health [4]. The maintenance of elevated energy consumption requires the uptake of glucose, amino acids, and lipids as energy substrates. Numerous lipids have been shown to have increased uptake into brown and beige adipocytes with cold exposure including free fatty acids, acylcarnitines, and lipoprotein complexes [5]. Beyond their direct role in fueling thermogenesis, lipids are important for their capacity as signaling molecules, components of membranes, and as posttranslational modifications [6]. These dynamic roles for lipids highlight their molecular complexity and the shift in lipid abundance as a measure of stored energy availability.

At the heart of thermogenic regulation and lipid processing is the mitochondrion, which is the site of the UCP1 function and lipid catabolism through β-oxidation. Mitochondria are highly abundant in brown and beige adipocytes and take on distinct morphology and inter-organelle interactions upon cold stimulation [7]. The scope of this review will focus on lipids that alter mitochondrial morphology or oxidative capacity, or that are produced in mitochondria. These lipids can be produced directly in brown or beige adipose tissue or in peripheral tissues including the liver and white adipose tissue.

## 2. Activation of Thermogenesis Increases Mitochondrial Fatty Acid Oxidation in Brown and Beige Adipocytes

Although brown and beige adipocytes both undergo thermogenesis, they are distinct in their cellular origin, localization, and mechanisms of thermogenesis [8]. Brown adipocytes are derived from myogenic factor 5 (Myf5) positive precursor cells that express PR domain containing 16 (PRDM16) and early B cell factor 2 (EBF2) [9,10]. In mice, brown adipose tissue (BAT) is found in the intrascapular region between the shoulder blades, while in humans it is found in the supraclavicular region and along the spinal cord. In contrast, beige adipocytes most commonly arise from Myf5 negative precursors that are Sca-1-positive; they can also be derived from transdifferentiation of white adipocytes. Some cases of Myf5 positive beige adipocytes have also been observed using Myf5 cre lineage tracing with reporter mice [11]. In mice, beige adipocytes are found in the subcutaneous adipose tissue after prolonged cold exposure or treatment with β_3_-adrenergic receptor (β_3_AR) agonist, although sex and strain differences in cellular distribution have been observed [12,13]. The presence of beige adipose tissue in humans is a source of contention. RNA-sequencing analysis showed human brown adipocytes clustering with mouse beige adipocytes and that chronic cold acclimatization led to thermogenic adipose tissue expansion into subcutaneous adipose tissue depots [14]. However, other work has shown that markers of beige adipose tissue such as Cd137, Tbx1, and Tmem26 are present in mouse brown adipose tissue with a high fat diet and thermoneutrality [15]. Regardless of cellular identity, these thermogenic adipose tissue depots significantly contribute to energy homeostasis in mice and humans, regulating body weight, glucose levels, and circulating lipids.

Upon cold exposure, the mitochondrial abundance of brown and beige adipocytes increases and the morphology, inter-organelle interaction, and protein composition shifts. The mitochondria in cold exposure have a spheroid morphology driven by increased fission. Norepinephrine stimulation activates protein kinase a (PKA) which phosphorylates dynamin-related protein 1 (DRP1) on serine residue 600 [7]. DRP1 activation leads to an accumulation of mitochondria, increased fission, and higher sensitivity of the mitochondria to free fatty acids. There is also decreased fusion with norepinephrine due to inactivation of the mitochondrial dynamin-like GTPase, optic atrophy protein 1 (Opa1), through cleavage to the less active short form [7]. With cold exposure, mitochondria also have decreased contact sites with lipid droplets, which leads to increased rates of respiration and fatty acid oxidation [16]. Finally, prolonged cold exposure alters brown adipocyte mitochondrial protein abundance, and proteomics revealed increased proteins in ubiquinone biosynthesis, fatty acid oxidation, and the tricarboxylic acid (TCA) cycle. There was also an upregulation of enzymes involved in glycerophospholipid synthesis including cardiolipin synthase, phosphatidylserine decarboxylase, and several acyltransferases [13,17]. In beige adipocytes, mitochondrial proteomics demonstrated that cold exposure increased arginine/creatine and proline metabolism, which revealed a novel mechanism of thermogenesis through phosphocreatine futile cycling [13]. Together, these observations reveal that cold exposure shifts mitochondria morphology in thermogenic adipocytes leading to increased fatty acid oxidation and lipid processing.

The increase in fatty acid oxidation and lipid processing is driven in part by a higher abundance of free fatty acids. In response to β_3_-adrenergic receptor (β_3_AR) activation, the white adipose tissue has increased lipolysis leading to elevated circulating free fatty acids (FFAs). These FFAs are processed by the liver into various species including triglyceride rich lipoproteins and acylcarnitines that enter the circulation and are taken up by brown and beige adipocytes. Upon entry into thermogenic adipocytes, FFAs bind UCP1, leading to activation. β_3_AR activation of thermogenic adipocytes also increases the response of adipocytes to FFAs. The treatment of brown adipocytes with norepinephrine and palmitate leads to increase mitochondrial depolarization and fatty acid oxidation beyond the addition of palmitate alone [7].

The elevated fatty acid oxidation and lipid uptake of thermogenic adipocytes has a major impact on whole body metabolism. Cold exposure increases the influx of lipids into brown adipose tissue by 12-fold [18]. Use of the apolipoprotein E knockout mouse, a model of hypertriglyceridemia, showed that cold exposure was able to normalize circulating free fatty acids and triglycerides [18]. In humans, the presence of BAT is correlated with circulating phosphatidylethanolamine and diacylglycerol, while also being negatively associated with dyslipidemia, type 2 diabetes, and cardiovascular disease [19,20]. Due to the regulation of systemic metabolism by mitochondrial lipid processing, signaling, and uptake in thermogenic adipocytes, a major focus of metabolic research has been to assess the diversity of lipids that are altered in cold exposure.

## 3. Lipids Produced by Brown and Beige Adipocytes

**Figure 1:**
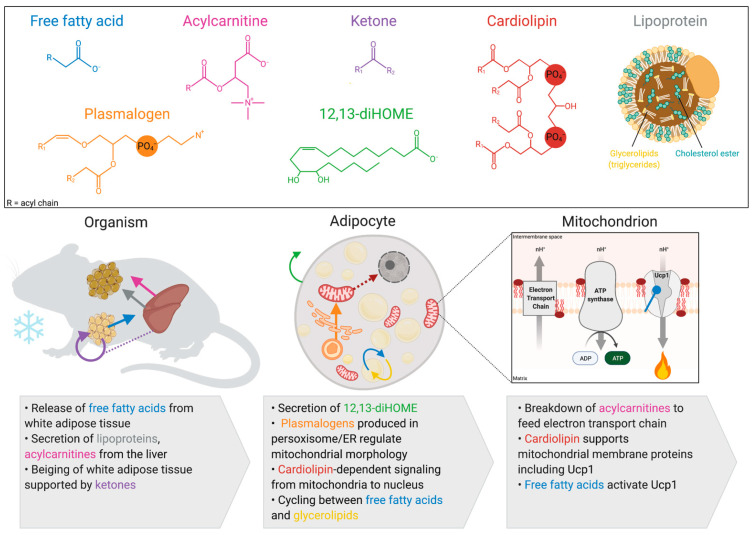
Lipids that regulate mitochondria to support thermogenesis in brown and beige adipocyte. Thermogenesis involves multi-organ orchestration of lipid processing. White adipocyte lipolysis releases circulating free fatty acids (blue arrow) which are taken up by the liver and thermogenic adipose tissue. Once in the liver, free fatty acids can be processed into lipoproteins (grey arrow) or acylcarnitines (pink arrow), which are released into the circulation to be taken up by brown adipose tissue. Some lipids are produced directly in the brown or beige adipocyte including 12,13-diHOME (green arrow), ketones (purple arrow), plasmalogens (orange arrow), and cardiolipin (red arrow). Many of these lipids have numerous roles including cardiolipin, which is a component of mitochondrial membranes, stabilizes uncoupling protein 1 (UCP1), and signals to the nucleus regulating transcription. Created with BioRender.

Lipid metabolism in brown and beige adipocytes shifts with cold exposure. There are increases in de novo lipogenesis with the activation of β_3_AR and thyroid hormone signaling as well as a complete restructuring of organelle contact sites and mitochondrial abundance [7,21,22]. This cellular restructuring leads to an altered lipid composition of brown and beige adipocytes [21]. We are limiting the scope of this review to lipids that directly impact mitochondrial function in thermogenic adipocytes. Focusing on lipids that are produced in brown and beige adipocytes, these lipids include cardiolipin, 12,13-dihydroxy-9z-ocatadecenoic acid, and plasmalogens (Figure 1).

### 3.1. Cardiolipin

Cardiolipin (CL) is a phospholipid found ubiquitously in the mitochondrial membranes of eukaryotic cells as well as in the membranes of many bacterial species. Mitochondrial function depends on CL, which has diverse roles in regulating membrane dynamics and morphology, protein interactions and activity, and mitochondrial signaling [22,23]. CL is composed of two phosphate headgroups and four acyl chains, giving it a conical shape that supports negative membrane curvature [24]. Maintaining points of negative curvature is essential for organizing the inner mitochondrial membrane into cristae structures, which provide optimal surface area for ATP-coupled and uncoupled respiration [25]. In addition to supporting membrane morphology, CL can regulate the conformation, complex assembly, and activity of integral membrane proteins, including the respiratory chain complexes [26]. CL also drives the membrane association of matrix proteins involved in essential respiratory processes like ubiquinone biosynthesis [27]. The exact function of CL is influenced by the length and degree of unsaturation of its acyl chains. After its initial synthesis, CL can undergo remodeling, which generally involves swapping in acyl chains that are longer and have more double bonds. Abnormalities in CL remodeling can have pathological consequences for multiple systems in the body, and is the basis for the genetic disorder Barth Syndrome, which commonly presents with cardiomyopathy and skeletal muscle weakness [28].

CLs are important in mitochondrial function and as signaling molecules during thermogenesis. CL synthesis increases with extended (>3 days) cold exposure in brown and beige adipose tissue, leading to an accumulation of specific CL species that may have distinct functions [29,30]. The general function of CL in supporting the respiratory chain is necessary to replenish the proton gradient that is dissipated by UCP1 to produce heat. CL may also directly interact with UCP1 to regulate its function, as it co-purifies with UCP1 with a predicted stoichiometry of three molecules of CL per UCP1 monomer [31]. This interaction improved the thermal stability of UCP1 in vitro, increasing its melting temperature (at which 50% of the protein is unfolded) by approximately 25 °C [31]. The functional role of cardiolipin binding to UCP1 in vivo is not yet known. Potentially, CL could provide tertiary stability, regulate assembly with other polypeptides, or influence a matrix-vs-cytosolic-facing conformation, as was shown for the related ADP/ATP carrier protein [32].

Beyond direct interaction with UCP1, CLs may also be involved in transcriptional regulation of Ucp1 and other thermogenic genes. Ablating CL production in adipocytes through the deletion of cardiolipin synthase (Crls1) resulted in decreased Ucp1 expression and cold sensitivity. Furthermore, overexpression of Crls1 was sufficient to increase Ucp1 expression and uncoupled respiration in vitro [29]. While it is possible that the observed changes in nuclear gene expression are a compensation for mitochondrial function being altered in general, these results support a model in which the nucleus can detect and respond to CL levels. The ER-stress response factor CHOP-10 was shown to mediate the downregulation of Ucp1 mRNA levels during CL deficiency; however, it is not known what mediates CL-dependent Ucp1 upregulation during adaptive thermogenesis [29]. Additionally, whether CL itself would be sensed in this model is unclear. CL can translocate to the outer mitochondrial membrane, making it available to interact with other organelles; this is observed during the breakdown of damaged mitochondria by mitophagy [33]. However, CL-dependent signaling may also be mediated by a protein that it interacts with. For example, disrupted interaction between CL and cytochrome *c* leads to the externalization of cytochrome *c* to trigger apoptosis [34]. More work is required to elucidate the mechanism of CL-based organelle crosstalk in brown adipose tissue.

### 3.2. 12,13-diHome

12,13-dihydroxy-9z-ocatadecenoic acid (12,13-diHOME) is produced when linoleic acid is oxidized to 12,13-epoxyoctadecenoic acid (12,13-epOME) by cytochrome P450, then 12,13-epOME is processed into 12,13-diHOME by soluble epoxide hydrolase (sHE or Ephx1/2). Originally, diHOME production was thought to be solely a toxic biproduct of epOME processing, because sHE over-expression induces cellular death and treatment of rat pulmonary alveolar epithelial cells with diHOMEs caused increased permeability and loss of epithelial integrity [35,36]. These observations are supported by the deletion of sHE in mice, which protects against inflammation-induced cardiotoxicity [37]. More recently, it has been appreciated that 12,13-diHOMEs also function as signaling molecules that regulate lipid homeostasis in exercise and thermogenesis [38].

Brown adipocytes increase the production and secretion of 12,13-diHOME with cold exposure in both mice and humans [39,40]. Activation of β_3_AR leads to the increased production of sHE transcripts and subsequent increases in 12,13-diHOME levels. This lipid can act as an autocrine or paracrine signal to induce the update of FFAs for oxidation in the mitochondria [39]. This increase in FFA uptake is driven through increased translocation of fatty acid transport protein 1 (FATP1) and CD36 to the plasma membrane [39]. The exact mechanisms by which 12,13-diHOME functions to increase FATP1 and CD36 translocation is unknown, and more work is needed to understand the molecular regulation.

The treatment of numerous cells including brown adipocytes, C2C12, and cardiomyocytes with 12,13-diHOMEs increased mitochondrial respiration and basal oxygen consumption rate [39]. In humans, 12,13-diHOME levels correlate with whole body metabolism: in a population study of 2248 individuals, plasma 12,13-diHOME was negatively correlated with adiposity, hyperlipidemia, and insulin resistance [41]. Although 12,13-diHOMEs are increased in BAT with cold exposure, other tissues also contribute significantly to the circulating pool, and the ablation of BAT does not alter circulating 12,13-diHOME levels [39]. Increased 12,13-diHOME secretion has been observed to occur in the skeletal muscle in response to exercise [38]. The relative contribution of each tissue to the 12,13-diHOME pool will be an important focus for future research.

### 3.3. Plasmalogens

Plasmalogens are glycerophospholipids that contain an ether-linked alkenyl chain in the sn1 position and an ester-linked fatty acid in the sn2 position. The synthesis of plasmalogens begins in the peroxisomes, and further processing continues in the endoplasmic reticulum. Plasmalogens are found in membranes throughout the cell including in the mitochondria, endoplasmic reticulum, and plasma membrane. Functionally, they are thought to serve as an antioxidant, and their abundance is high in cells sensitive to oxidative damage including neurons, cardiomyocytes, and skeletal muscle as well as brown and beige adipocytes. The double bond of the alkene chain is susceptible to oxidation and is rapidly turned over, preserving the other phospholipids from peroxidation [42]. Plasmalogens have also been shown to function as a signaling molecule regulating ferroptosis [43].

In brown and beige adipocytes, plasmalogens regulate mitochondrial morphology. The activation of PRDM16 increases peroxisomal proliferation during cold exposure through transcriptional regulation of peroxisomal proteins, and targeted ChIP-qPCR showed PRDM16 occupancy in the promoter of peroxisomal proteins including Pex16 [44]. The knockout of Pex16 in adipose tissue (Pex16 AKO) led to mitochondria with fused morphology, impaired thermogenesis, decreased peroxisomes, and a subsequent decrease in plasmalogens. Dietary supplementation of plasmalogen precursors alkylglycerols rescued plasmalogen levels, mitochondrial morphology, and cold sensitivity in Pex16 AKO mice [5,44]. Furthermore, a knockdown of glyceronephosphate O-acyltransferase, a peroxisomal enzyme that regulates plasmalogen synthesis, led to impaired mitochondrial fission and an ablated oxygen consumption rate in isolated brown adipocytes. Together, these studies suggest an important role for plasmalogens in thermogenesis, although more work is needed to determine the mechanism through which plasmalogens regulate mitochondrial morphology.

## 4. Inter-Organ Lipid Signaling from White Adipose Tissue

### 4.1. Free Fatty Acids

Free fatty acids (FFAs) are carboxylic acids with acyl-chains of various lengths and desaturation. In brown and beige adipocytes, several pathways involved in FFA metabolism are simultaneously upregulated in response to cold including de novo lipogenesis, lipolysis, and oxidation [45,46]. The lipolysis of FFAs in response to cold begins when β_3_AR activation increases cAMP concentrations to activate PKA. Lipolysis occurs in several steps, beginning with triglyceride hydrolysis by adipose triglyceride lipase (ATGL), which forms diacylglycerol. Diacylglycerol is then hydrolyzed by hormone sensitive lipase (HSL) to form monoacylglycerol, which is broken down into an FFA and glycerol by monoacylglycerol lipase (MGL). The activation of lipolysis by β_3_AR activation occurs in brown, beige, and white adipocytes, but recent work has demonstrated that brown and beige adipocytes are reliant on FFAs released from white adipocytes for thermogenesis. Whole-body or adipose tissue-specific KO of ATGL leads to severe cold sensitivity that is fatal within 90 min of cold exposure [47,48,49]. However, loss of ATGL in thermogenic adipocytes driven by UCP1-cre has no impact on thermogenesis, suggesting that it is white adipocyte lipolysis that drives the brown adipocyte pool [47]. These observations are further supported by the knockout of diacylglycerol acyltransferase 1 and 2 (DGAT 1 and 2) using the UCP1-cre driver, which had no impact on thermogenesis, even though the mice lacked lipid droplets in their brown adipocytes [50].

Once FFAs enter brown and beige adipocytes there are several mechanistic roles to support thermogenesis including serving as a fuel substrate for β-oxidation and direct binding to UCP1 to regulate protein function and conformation. FFA binding to UCP1 is essential for facilitating the transport of protons across the mitochondrial membrane. Recent patch-clamp studies support a mechanism by which a long chain FFA is bound to UCP1, flipping its head group between the matrix and inner membrane space, binding and releasing protons in a pKa dependent manner [51]. Structural studies using NMR bolster this model by determining that K56 and K269 residues of UCP1 bind FFAs through electrostatic interaction to allow for shuttling of both FFAs and protons through UCP1.

Beyond their requirement in β-oxidation and as a UCP1 binding factor in thermogenic adipocytes, FFAs are also known to be central players in glycerolipid/free fatty acid futile cycling, which involves the continuous anabolism and catabolism of glycerolipids (GLs) to generate heat [52,53]. During anabolism, the energy of the thioester bond of fatty acyl-CoAs makes the ester bond between the hydroxyl group of the glycerol and the fatty acyl. As each ester bond of triacylglycerol is hydrolyzed, the energy of the bond is dissipated as heat. This model is challenged by the lack of cold sensitivity in UCP1-cre driven KO of ATGL in mice [47]. However, these GLs can be diverse, including any lipid with a glycerol backbone, including triglycerides, diacylglycerides, monoacylglycerides, and phospholipids. Moreover, GL/FFA cycling can also involve whole body cycling, where free fatty acids are released from white adipocytes and triglycerol is built in skeletal muscle, hepatocytes, or brown and beige adipocytes [52]. The constant breakdown and release of FFAs from adipocytes has been observed in fasting in rats, mice, and humans, with 40% of FFAs being rapidly recycled back to triglycerides [54]. More recently, it has been shown that β_3_AR signaling blocks the re-esterification of FFAs to TGs, potentially allowing for an inter-organ GL/FFA cycle [55].

### 4.2. Ketones

Ketones are a lipid-derived metabolite that include acetoacetate, β-hydroxybutarate, and acetone. Ketones serve as an important fuel source in thermogenesis, but also function as a signaling molecule to drive differentiation of beige adipocytes. This signaling role was discovered by Wang et al. (2019), through the use of a media transfer between beige adipocytes and preadipocytes. This work demonstrated that PRDM16 drives fatty acid oxidation and ketone production in beige adipocytes, and the transfer of this conditioned media to adipocyte precursors enhanced beige adipocyte differentiation [56]. The treatment of these adipocyte precursors with β-hydroxybutarate alone was able to recapitulate this phenotype. A loss of ketone production by the knockout of 3-Hydroxybutyrate Dehydrogenase 1 (Bdh1) led to increased fibrosis and decreased beige adipocyte differentiation in response to cold acclimatization [56]. These findings may explain previous observations that ketogenic-diet-fed mice had increased mitochondrial proteins, UCP1, oxygen consumption, and improved thermoregulation [57,58]. There are also several studies that demonstrate that circulating ketones are elevated in response to cold exposure, including a human study that placed males in a 0 °C room for 90 min. In this study, ketones in the plasma increased when the subjects were exposed to the cold while urinary excretion of ketones decreased [59]. Together, these studies suggest that ketones regulate thermogenic potential by expanding the beige adipocyte population and increasing respiration in brown adipocytes. Further work will be needed to determine the contribution of various tissues to the elevated levels of circulating ketones in cold exposure.

## 5. Inter-Organ Lipid Signaling from the Liver

### 5.1. Lipoprotein Complexes

Instead of being taken up by brown and beige adipocytes, FFAs from white adipocyte can also be taken up by the liver, where they are processed into triglycerides, lipoprotein complexes, and acylcarnitines. Lipoproteins consist of a core of cholesterol esters and triglycerides surrounded by a surface monolayer of cholesterol, phospholipids, and apolipoproteins. Because cholesterol and triglycerides are insoluble in the blood, these complexes allow for ferrying of these complex lipid species between tissues. Plasma lipoproteins have several classes; those that are synthesized in the liver include very low density lipoprotein (VLDL), low density lipoprotein (LDL), and high density lipoprotein (HDL). In cold exposure, hepatic triglycerides and VLDL production is increased as is secretion of VLDL particles [60]. These lipoprotein complexes are taken up by the brown adipose tissue during cold exposure or β_3_AR agonist, as demonstrated by the radioactive labeling of triglycerides and cholesterol esters [18,61]. Similar observations are seen in humans, where despite the cold-induced increased lipoprotein complex production, the elevated uptake by brown adipose tissue leads to improved circulating lipid profiles [18,62]. Other work in humans found that detectable BAT correlates with lower plasma cholesterol and LDL, and prolonged daily cold exposure of 20 min per day for 90 days decreased total cholesterol, LDL, and hemoglobin A1C [63].

### 5.2. Acylcarnitines

Acylcarnitines are made from fatty acids esterified to carnitine molecules. At the cellular level, acylcarnitines are known to be an intermediate product of FFA transport. They are produced on the outer surface of the mitochondrial membrane by carnitine palmitoyl transferase 1 (CPT1) to facilitate the transport of long chain fatty acids (LCFA) across the mitochondrial membrane for breakdown by β-oxidation. Incomplete β-oxidation can lead to an accumulation of acylcarnitines within the cell as well as in the circulation. Serum acylcarnitines correlate with diet-induced obesity, insulin resistance, and diabetes in mice [64]. In humans, total plasma acylcarnitines are increased in diabetic compared to nondiabetic plasma, driven in part by high levels of short chain (2–6 carbons) and medium chain (6–12 carbons) acylcarnitines [65]. In nondiabetic men, plasma medium chain acylcarnitines correlated with worsened glucose tolerance test [66]. These findings have led to the suggested use of acylcarnitines as biomarkers for metabolic syndrome.

In the circulation, acylcarnitines have also been identified as an important energy source for brown fat thermogenesis [67]. In mice, the production of acylcarnitines in hepatocytes increases during cold exposure. This increased hepatic production of acylcarnitines is driven by FFA lipolysis from the white adipose tissue, adipocyte-specific KO of ATGL ablated hepatic acylcarnitine production in response to β_3_AR activation. In cold exposure, these circulating acylcarnitines are taken up by BAT, skeletal muscle, and the heart. Once they are taken up by brown adipocytes, acylcarnitines are broken down. The use of heavy labeled ^13^C-palmitoylcarnitine in cultured brown adipocytes stimulated with β_3_AR agonist showed the incorporation of label into TCA cycle intermediates, indicating that acylcarnitines were broken down as a fuel source in the mitochondria. Despite these findings, it is still unclear if acylcarnitines act solely as a fuel source or if they have other functions in supporting BAT thermogenesis.

More work is needed to understand the impact of circulating acylcarnitines in human thermogenesis. Interestingly, a variant of CPT1A, the predominant CPT1 isoform in the liver, is found in Inuit populations in Greenland, Alaska, and Canada [68]. This polymorphism is a proline to leucine substitution at 479, which is in the region of the protein that facilitates malonyl-CoA inhibition causing CPT1A to always be active, even during conditions of high glucose when fatty acid oxidation should be decreased [68,69]. The leading theory for the high prevalence of this variant is that it is adaptive to the consumption of traditional Inuit foods, which are high in fat and protein but low in carbohydrates [68]. Recent studies have supported this theory, with the polymorphism being associated with diet and circulating omega-3 fatty acids [70]. Several other proteins in the acylcarnitine processing pathway such as carnitine-O acetyltransferase (CrAT), CPT1B, and CPT2 also have variants that are frequent in the Inuit population [71]. The impact of this variant on adaptive thermogenesis has yet to be explored.

Acylcarnitines also actively impact cellular signaling and inter-organ communication [72]. One example of this secondary role is found in the stimulation of the inflammatory response by acylcarnitines. In cultured mouse monocytes, medium chain acylcarnitines induced signaling of the NF-κB (nuclear factor kappa-light-chain-enhancer of activated B cells) inflammatory pathway [65]. Acylcarnitines may also impact insulin signaling in the body [73]. The knockout of malonyl-coenzyme A decarboxylase in mice led to partial inhibition of CPT1 and, as a result, lower levels of acylcarnitines; these mice exhibited greater levels of insulin sensitivity [64]. This observation suggests that the production of acylcarnitines and incomplete β-oxidation contributes to insulin resistance. One proposed mechanism in which acylcarnitines impact insulin signaling is inhibiting phosphorylation of the protein kinase B (AKT), a downstream component of the insulin signaling cascade. Treating muscle cells with palmitoylcarnitine or with separated fatty acid and carnitine reduced the phosphorylation of AKT [64,74]. This indicates that acylcarnitines impact insulin signaling in skeletal muscle; however, whether this applies to BAT is not yet known. It is still uncertain if insulin signaling and inflammation are the only signaling cascades in which acylcarnitines play a role.

## 6. Perspectives

Thermogenesis in brown and beige adipocytes is dependent on mitochondrial lipid processing. These lipids can be produced directly in brown and beige adipocytes or can come from peripheral sources including white adipocytes or the liver. Lipids generated in the brown and beige adipocytes that alter mitochondrial function include CLs, 12,13-diHOMEs, and plasmalogens. Peripherally produced lipids and lipid complexes enter the circulation and are taken up by thermogenic adipocytes including acylcarnitines and triglyceride-rich lipoproteins [18,67]. Other lipids have numerous sources including FFAs and the lipid-derived metabolite ketones. FFAs primarily come from white adipose tissue lipolysis but can also come from brown and beige adipocytes. Many lipids have several roles, such as cardiolipins that provide structural support to mitochondrial membranes, stabilize UCP1, and signal to the nucleus for transcriptional regulation [30]. Similarly, ketones are an important fuel substrate but also regulate beige adipocyte differentiation. The ability of lipids to play numerous roles highlights the dynamic nature of these molecules and emphasizes the need to reassess our limited view of lipids as single purpose molecules.

We have limited the scope of this review to focus on lipids that are produced by or impact mitochondria in the brown and beige adipocytes. There are several lipids that impact thermogenesis that were left out due to this narrow designation including sphingolipids, dolichols, diacylglycerols, prostaglandins, and 12-hydroxyeicosapentaenoic acid (12-HEPE) [75,76,77]. Some of these lipids function through mechanisms that impact mitochondria only in a secondary manner such as 12-HEPE, which activates G-protein coupled receptors to increase glucose uptake in brown adipocytes [77]. Other lipids potentially have a role in mitochondrial regulation, such as sphingolipids and ceramides, but the exact mechanism of this action is yet to be understood. Recent work demonstrated that a UCP1-cre mediated knockout of serine palmitoyltransferase subunit 2, an enzyme important in ceramide synthesis, led to increased mitochondrial density, while knockout of an enzyme in ceramide degradation led to reduced mitochondrial density [78]. Further exploration into the mechanisms by which ceramides are driving these mitochondrial differences is needed. Another subset of these lipids impacts the conversion of white adipocytes to beige adipocytes such as prostaglandin H2, but the direct regulation of mitochondria function and thermogenesis is unexplored [6]. The importance of prostaglandins in thermoregulation warrants further investigation, but their known regulation of body temperature for fevers is intriguing.

Physiological factors such as sex or age influence the lipid composition of brown adipocytes. The lipidomic analysis of BAT from female or male mice revealed sex-specific differences in phospholipid acyl chains, with more incorporation of stearic and arachidonic acid in females, and palmitic and linoleic acid in males [79]. Increased desaturation of mitochondrial phospholipids impacts membrane dynamics and may underly the dimorphism in the mitochondrial size and shape observed between male and female BAT in rats [80]. Aging also alters BAT lipid metabolism. In the BAT of aged mice, decreased production of lipoic acid leads to suppression of catabolic pathways including fatty acid oxidation [81]. It was also seen that as mice age, their capacity to regulate body temperature during cold exposure is limited because of reduced acylcarnitine production in the liver. When acylcarnitines were administered to aged mice during cold exposure, BAT thermogenesis improved [67]. How lipid-based signaling in BAT is impacted by sex and age requires further study.

More work is needed to understand lipids that impact mitochondria in beige adipocytes. This is difficult because the emergence of beige adipocytes in subcutaneous adipose tissue is heterogeneous and occurs in pockets surrounding vasculature [82]. Moreover, the advent of single cell and single nuclei RNA sequencing, as well as the refinement of cold stress conditions, have demonstrated that there are numerous subtypes of beige adipocyte that have differences in glycolytic capacity and cellular origin [83,84,85,86]. These studies have also revealed lipid signaling between beige adipocytes and resident macrophages that regulates the thermogenic response [84,87]. The advent of single cell metabolomics coupled with cell sorting will enable the exploration of the lipid composition of individual subtypes of beige adipocytes [88].

At the cellular level, several emerging technologies have led to higher lipid visualization and quantitation. Mass-spectrometry-based lipidomics has unearthed previously unidentified lipids including signaling molecules such as fatty acid esters of hydroxy fatty acids (FAHFAs), which regulate insulin sensitivity [89,90]. Chemical probes including photoswitches have the capacity to functionally characterize lipids and the proteins they interact with, while photocleavable groups can facilitate the temporal range of lipid activity [91]. Labels including fluorescent tags such as Bodipy and GFP as well as luminescent tags on acyl-chains provide imaging potential to determine cellular localization and lipid uptake [92,93,94]. Further tools are needed to increase the capacity to track lipid mobility and uptake in vivo to determine novel inter-organ communication pathways. Currently, quantitative assessment of lipid mobility is through radioactive or heavy isotope labeling. Radioactivity is sensitive and can be used to assess lipid uptake from the circulation and quantitatively assess oxidation but can be difficult to use in vivo. Heavy isotope labeling is cost prohibitive in vivo and the expertise for the quantitative calculation of pathway input is limited to several labs around the world [95,96]. Both technologies are limited in their ability to assess inter-organ signaling pathways. As these tools are developed and applied in tandem, they will expand our depth of understanding for the importance of lipid metabolism in thermogenic adipose tissue.
